# Protocol for *in situ* transplantation of adipose stem cells into mouse epididymal white adipose tissue

**DOI:** 10.1016/j.xpro.2025.104035

**Published:** 2025-08-15

**Authors:** Xiyan Liao, Qin Zeng, Haowei Zhang, Limin Xie, Tuo Deng

**Affiliations:** 1 Division of Endocrinology, Department of Internal Medicine, The First Affiliated Hospital of Zhengzhou University, Zhengzhou 450052, China; 2 Department of Clinical Nutrition, The Third Xiangya Hospital, Central South University, Changsha, Hunan 410013, China; 3 The First Affiliated Hospital, Department of Orthopedics, Hengyang Medical School, University of South China, Hengyang, Hunan 421001, China; 4 National Clinical Research Center for Metabolic Diseases and Department of Metabolism and Endocrinology, The Second Xiangya Hospital of Central South University, Changsha, Hunan 410011, China

**Keywords:** Cell isolation, Metabolism, Stem Cells

## Abstract

Adipose stem cells (ASCs) play a crucial role in maintaining adipose tissue homeostasis, but studying them *in situ* is technically challenging. Here, we present a protocol for the transplantation of ASCs into mouse epididymal white adipose tissue (eWAT). We describe steps for isolating and purifying ASCs, labeling them with a cell tracker, administering anesthesia, making an incision, and performing spot injections of ASCs into the eWAT of mice. This protocol offers broad applications for *in vivo* functional research of ASCs.

For complete details on the use and execution of this protocol, please refer to Liao et al.^1^

## Highlights

• Details on the isolation and purification of ASCs from high-fat diet-fed mice
• Instructions for labeling ASCs with a cell tracker and resuspending in Matrigel
• Procedures for spot injection of ASCs into mouse eWAT

## Abstract (editor highlights)

Publisher’s note: Undertaking any experimental protocol requires adherence to local institutional guidelines for laboratory safety and ethics.

## Graphical abstract

**Figure:**
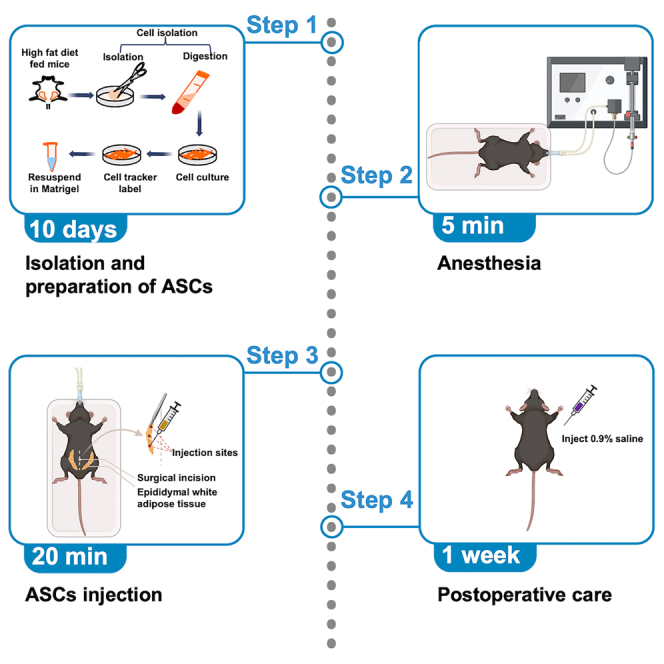

## Before you begin

The following protocol outlines the detailed steps for transplanting ASCs into eWAT. We isolate ASCs, perform surgery and inject ASCs into the fat pad. To improve the survival rate of transplanted ASCs, we inject them at three different spots during the needle withdrawal process. This protocol was applied to investigate the role of ASCs in obesity-induced adipose tissue inflammation.^1^ ASCs are heterogeneous, this protocol can also be applied to investigate the characteristics of ASC subpopulations in vivo.

### Institutional permissions

Appropriate institutional approvals for Biosafety and use of animals should be obtained before starting this protocol. All animal protocols are approved by the Institutional Animal Care and Use Committee of the Central South University.

### Preoperative preparation

**Timing: 1 day (day −1; 1 day before the surgery)**

**Figure 1:**
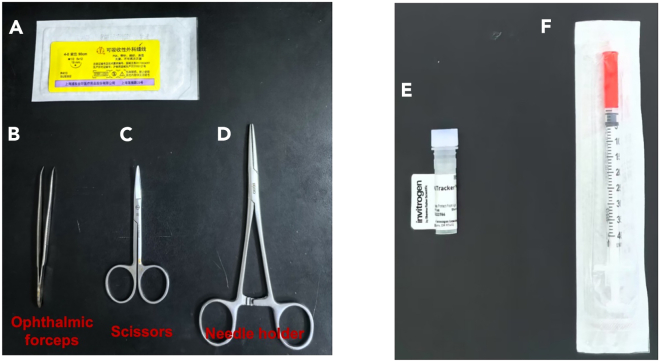
Instruments used in this protocol (A) Surgical sutures. (B) Ophthalmic forceps. (C) Scissors. (D) Needle holder. (E) Cell tracker. (F) Syringe for ASCs.

This section outlines the equipment and materials necessary for the surgery (Figure 1).

1. Place the Matrigel at 4°C for 12–16 h before the injection, and chill the tips and syringes at −20°C for at least 30 min.

**CRITICAL:** Ensure that the Matrigel, tips, and syringes are kept on ice throughout the procedure.

**Figure 2:**
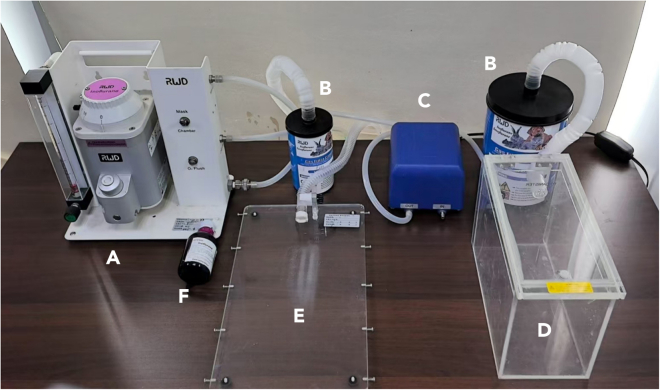
The anesthesia-ventilator system (A) Anesthesia machine. (B) Gas filter. (C) Air pump. (D) Anesthesia chamber. (E) Operating table. (F) Isoflurane solution.

2. Sterilize surgical instruments using an autoclave (Figures 1B–1D).
3. Prepare the isoflurane anesthesia system:
  a. Properly install the anesthesia system, including the anesthesia machine, gas filter, air pump, anesthesia chamber, and operating table (Figure 2).
  b. Add the isoflurane solution (100%) to the anesthesia machine.
  c. Properly set the anesthesia system parameters (detailed in the materials and equipment setup).

**CRITICAL:** Appropriate anesthesia system settings are crucial for ensuring effective anesthesia and the safety of mice during surgical procedures.

4. Mice preparation:
  Male C57BL/6J mice are housed under specific-pathogen-free (SPF) conditions with a 12-h light-dark cycle.
  a. 6-week-old male C57BL/6J mice are fed a high-fat diet consisting of 60% kilocalories from fat (Research diets Inc., D12492) for 12 weeks. After 12 weeks of high-fat feeding, the mice (38–42 g) are used for ASCs isolation.
  b. 10-week-old male C57BL/6J mice (22–25 g) fed a standard chow diet are used for ASC injection.

## Key resources table

**Table:** 

REAGENT or RESOURCE	SOURCE	IDENTIFIER
**Chemicals, peptides, and recombinant proteins**		

DMEM	Gibco	Cat# 11330501CP
DMEM/F12	Gibco	Cat# 11330500CP
Fetal bovine serum	Gibco	Cat# 10099-141C
Penicillin-streptomycin	Gibco	Cat# 15140122
b-FGF	PeproTech	Cat# 100-18B
Phosphate-buffered saline	Invitrogen	Cat# AM9624
CellTracker	Invitrogen	Cat# C34565
TrypLE Express	Gibco	Cat# 12604039
Matrigel	Corning	Cat# 356237
Collagenase II	Worthington Biochemical	Cat# LS004177
Isoflurane solution	RWD Life Science	Cat# R510-22-10

**Experimental models: Organisms/strains**		

Mouse: C57BL/6J, 6 weeks, male	The Jackson Laboratory	N/A

**Other**		

R500 small animal anesthesia machine	RWD Life Science	Cat# R500IE
4–0 absorbable surgical sutures	Jinhuan Medical	N/A
Sterile single-use syringes for insulin (29G)	BD	Cat# 328421
Fine scissors sharp	Hunan far biological technology	Cat#YT141
Needle holder	Hunan far biological technology	Cat# YF2354
Ophthalmic forceps	Hunan far biological technology	Cat# YA1263
High-fat diet	Research Diets	Cat# D12492

## Materials and equipment

**Table:** Anesthesia system

Parameter	Setting
O_2_ flowmeter	1–2 L/min
Isoflurane concentration	2%–3%

## Step-by-step method details

### Isolation and purification of ASCs

**Timing: 50 min (for step 1)**

**Timing: 10 days (for step 2, day −10; 10 days before the surgery)**

This section outlines the steps of isolating and purification of ASCs from eWAT of high fat diet fed mice.

1. Isolation of ASCs from eWAT.
  a. Sterilize surgical instruments including fine scissors and ophthalmic forceps. Prepare 15 mL tubes, 75% alcohol, 100-μm cell strainer and DMEM.
  b. Set the incubator shaker temperature to 37°C.
  c. Prepare collagenase Ⅱ buffer: Dissolve collagenase in DMEM to a concentration of 1 mg/mL. Keep the collagenase Ⅱ buffer in water bath (37°C).
  d. Euthanize the mice by cervical dislocation and subsequently immerse them in 75% ethanol for 5 min.
  e. Put mice on a sterile laminar flow hood. Create a 1 cm longitudinal incision in the lower-mid abdomen of the mouse.
  f. Gently lift the epididymal fat pad through the incision using forceps, and carefully excise it with scissors.
    ***Note:*** Approximately 1.5–2.0 g of adipose tissue can be obtained from a single mouse.
  g. Cut the adipose tissue into approximately 1 cm^3^ pieces on ice using scissors.
  h. Transfer the tissue into collagenase Ⅱ buffer and incubate for 30 min at 37°C while shaking at 120 rpm.
    ***Note:*** The volume of collagenase Ⅱ buffer depends on the tissue volume; We use 5 ml of collagenaseⅡbuffer to digest 1 g adipose tissue.
  i. Filter the cells with a 100-μm cell strainer into a 15 mL tube.
  j. Centrifuge the cell suspension at 500 × g for 5 min at 4°C. A red pellet representing the stromal vascular fraction (SVF) will form at the bottom of the tube, with floating adipocytes at the top.
  k. Discard the supernatant by pouring it off. Resuspend the pellet in 10 mL of DMEM/F12 medium containing 10% fetal bovine serum (FBS), 1% Penicillin-Streptomycin (Pen/Strep), and b-FGF (10 ng/ml).
2. Culture SVF in DMEM/F12 medium containing 10% FBS, 1% Pen/Strap, and b-FGF (10 ng/ml). Passage the cells when they reach approximately 90% confluence. ASCs are purified by cell culture. The third passage cells are identified as ASCs.^2^

***Note:*** Approximately 8–10 × 10^6^ ASCs can be derived from a single mouse.

### ASC preparation

**Timing: 50 min**

This section outlines the steps of labeling ASCs with a cell tracker and resuspending ASCs in Matrigel.

3. Label ASCs using a cell tracker.
  a. Pre-warm DMEM containing CellTracker (1 μM) in a 37°C water bath for 10 min.
    ***Note:*** The CellTracker dye used in this protocol is Deep Red, with an excitation wavelength of 630 nm and an emission wavelength of 660 nm. For visualization, a microscope equipped with a 630–640 nm excitation source and a 650–670 nm emission filter is recommended. The dye can be detected using settings compatible with the Cy5 channel.
  b. When the third passage cells reach approximately 90% confluence, discard the DMEM/F12 medium, and wash the cells three times with phosphate-buffered saline (PBS).
  c. Incubate ASCs in 5 mL of DMEM containing 1 μM CellTracker in a cell culture dish at 37°C in a cell culture incubator for 30 min.
    ***Note:*** The volume of DMEM containing CellTracker used for incubating ASCs depends on the size of the cell culture dish. In this protocol, we culture ASCs in 10-cm cell culture dishes.
  d. Wash the cells three times with phosphate-buffered saline (PBS). Digest ASCs with 0.5 mL TrypLE Express for 1 min in a 37°C cell culture incubator, and terminate the digestion by adding 5 mL DMEM/F12 containing 10% FBS.
4. Resuspend ASCs in Matrigel.
  a. Mix DMEM and Matrigel at a 7:1 ratio and keep the mixture on ice.
  b. Perform cell counting and centrifuge the cell suspension at 500 × g for 5 min at 4°C. Then gently resuspend the cell pellet in a pre-chilled mixture of DMEM and Matrigel at a specified volume on ice.

***Note:*** The volume of the medium used for cell resuspension depends on the cell number. We resuspend 1 × 10^6^ cells in 100 μL of the DMEM and Matrigel mixture.

**CRITICAL:** It is important to gently resuspend the cells to avoid the formation of foam.

### ASC injection

**Timing: 25 min**

This section outlines the steps of anesthesia and surgical preparation, and ASCs injection.

**Figure 3:**
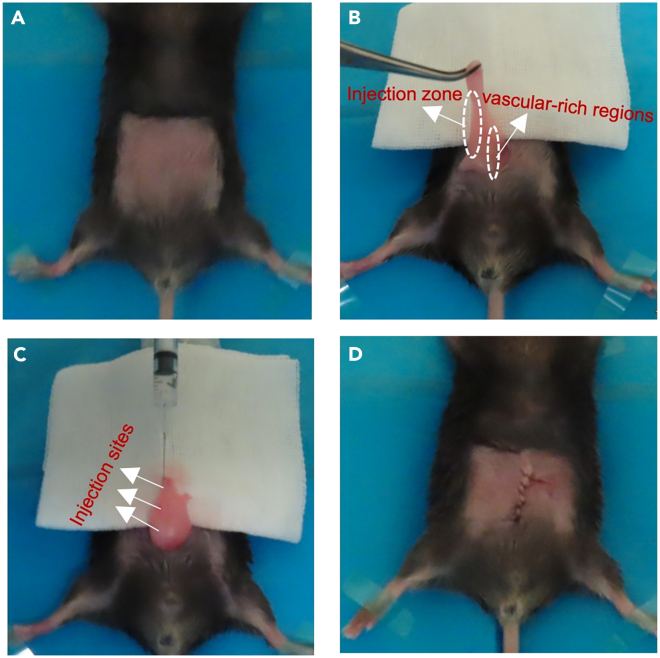
ASC injection (A) Disinfect the abdomen. (B) Gently lift one side of the adipose tissue through the surgical incision using forceps. (C) Insert the syringe along the blunter side of the adipose tissue and inject cells at three points during needle withdrawal. (D) Suture the abdominal incision in layers.

5. Anesthesia and surgical preparation.
  a. Turn on the anesthesia system and connect it to the anesthesia chamber.
    i. Set the oxygen flow rate on the flowmeter to a range of 1–2 L/min.
    ii. Set the isoflurane concentrator to a concentration between 2%–3%.
  b. Place each mouse in the anesthesia chamber.
  c. Verify deep anesthesia by gently applying pressure to the footpad.
    ***Note:*** The mouse will exhibit no response to stimulation if the deep anesthesia is successfully achieved.
  d. Put mice on the operating table. Secure the limbs with adhesive tape on the operating table and fully expose the mouse’s abdominal skin.
  e. Switch the anesthesia system to the ventilator.
  f. Activate the ventilator.
    ***Note:*** It is crucial to ensure that the mouse remains under anesthesia throughout the entire procedure.
6. ASCs injection.
  a. Thoroughly disinfect the abdomen using 75% ethanol solution (Figure 3A).
  b. Make an approximately 1 cm longitudinal incision in the lower-mid abdomen of the mouse with fine scissors sharp. Carefully cut through each layer of the abdominal wall, ensuring the incision passes through the skin and the rectus abdominis muscle troubleshooting 1.
  c. Prepare 50 μL ASCs in Syringe with 29G needles.
    ***Note:*** We inject 50 μL ASCs into each side of the fat pad. Some cells will waste in the needle, so always prepare more cells and more volume than you need.
  d. Locate and fully expose the right eWAT by performing blunt separation with an ophthalmic forceps (10 cm, curved with hook) (Figure 3B).
  e. Find the blunter side of the adipose tissue and insert the syringe along this side. Inject the cells at three points along the insertion path during the needle withdrawal (Figure 3C) troubleshooting 3.
    **CRITICAL:** It is crucial to inject the cells at three points during the needle withdrawal to improve the survival rate of transplanted cells.
  f. Hold the needle in the fat tissue for 3 s and slowly remove the needle.
    ***Note:*** Carefully remove the needle from the fat pad to prevent ASCs from flowing out of the tissue.
  g. Arrange and reposition the eWAT back into the abdominal cavity of mice with ophthalmic forceps.
  h. Perform the same procedure to inject ASCs into the left eWAT.
  i. Suture each layer of the abdominal wall of the mouse intermittently after injection using absorbable surgical sutures, ensuring no sutures are left to minimize the risk of infection (Figure 3D) troubleshooting 1.
    ***Note:*** A group size of 6–8 male mice are recommended to ensure reproducible results and statistical robustness.
    **CRITICAL:** Limit the surgery to a maximum of 20 min to prevent excessive body temperature loss. Adhering to this time limit is crucial for the animal’s well-being and optimal recovery. If the procedure exceeds this duration, a heating pad should be used to maintain the mouse’s body temperature and minimize the risk of hypothermia.

### Postoperative care

**Timing: 1 week (day 1 to day 7)**

7. Inject pre-warmed (37°C) 0.9% saline subcutaneously to prevent dehydration resulting from surgery.
8. Place the mouse in a warm and dimly lit environment until it fully regains consciousness.

***Note:*** After surgery, an optional analgesic regimen (e.g., buprenorphine) may be administered to ensure animal welfare.

9. After operation, place the mouse in a clean cage. Conduct daily observations to monitor for any signs of pain, difficulty breathing, or impaired movement.

## Expected outcomes

**Figure 4:**
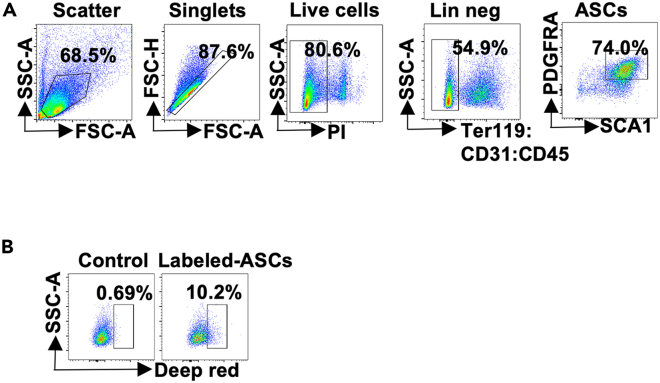
Survival rate of labeled ASCs (A) Gating strategy for ASCs (Pdgfrα^+^Sca1^+^ cells). (B) Flow cytometry plots of cell tracker labeled ASCs in Pdgfrα^+^Sca1^+^ cells from eWAT in mice transplanted with unconditioned control medium (Control) and cell tracker labeled cells. The control group is from mice not transplanted with labeled cells, which could be a negative control of the specific gates.

Upon disconnection from the ventilator, the mice resumed spontaneous respiration. Approximately 15–20 min after surgery, the mice regain consciousness with no impairment in motor activity. In our work,^1^ seven days post- transplantation, flow cytometry analysis demonstrated that more than 10% of the Pdgfra^+^ Scal1^+^ cells in the recipient mice eWAT were identified as labeled ASCs (Figure 4), indicating successful ASC transplantation. Mice transplanted with obese ASCs exhibited impaired insulin sensitivity and increased adipose tissue inflammation. This protocol has potential for investigating the immune and metabolic effects of ASC subpopulations in vivo.

## Limitations

Although our protocol is optimized to ensure reproducibility and reliability in our experiments, it is important to acknowledge its limitations due to the potential risks inherent in the surgical procedure. For example, the blood vessels at the site of syringe insertion may be compromised. Matrigel has been used as a scaffold for ASC transplantation.^3^ In our study, we optimize the DMEM-to-Matrigel ratio, identifying a 7:1 mixture as yielding the highest ASC survival rate. However, we do not assess other commonly used biomaterials, such as fibrin, alginate, or collagen gels, for their impact on ASC survival following transplantation. Nevertheless, the protocol can potentially be adapted to alternative extracellular matrix scaffolds, depending on material availability and experimental objectives.

## Troubleshooting

### Problem 1

After surgery, the mice experience mortality.

### Potential solution

Make moderate-sized incisions to minimize tissue damage.

Ensure that each layer of the abdominal wall is sutured properly.

Ensure the person performing the surgery possesses adequate training and expertise to reduce operation time.

### Problem 2

Wound infection in mice resulting from the surgical procedure (related to ASCs injection).

### Potential solution

Make sure all instruments are sterile and that the procedure is conducted in a sterile environment.

Ensure that the individual performing the surgery has the necessary training and expertise.

### Problem 3

The survival rate of injected ASCs is low.

### Potential solution

Keep the sterile BD insulin syringe inside the eWAT until the injection is complete, and inject the ASCs slowly to prevent leakage of ASCs into the abdominal cavity of the mouse.

## Resource availability

### Lead contact

Further information and requests for resources and reagents should be directed to and will be fulfilled by the lead contact, Tuo Deng (dengtuo@csu.edu.cn).

### Technical contact

Technical questions on executing this protocol should be directed to and will be answered by the technical contact, Xiyan Liao (xiyanliao@zzu.edu.cn).

### Materials availability

This study did not generate new unique reagents.

### Data and code availability

This study did not generate any unique datasets or code. The published article includes all datasets generated or analyzed during this study.^1^

## Acknowledgments

This work was supported by grants from the Henan Province Medical Science and Technology Research and Development Plan Joint Construction Project (LHGJ20240202), the Natural Science Foundation of Hunan Province (2023JJ40809), and the Natural Science Foundation of Changsha (kq2208318). The graphical abstract was created using Biorender.com.

## Author contributions

Conceptualization, X.L. and Q.Z.; methodology, X.L., Q.Z., and H.Z.; investigation, X.L. and Q.Z.; writing – original draft, X.L.; writing – review and editing, X.L., Q.Z., and L.X.; funding acquisition, X.L. and Q.Z.; supervision, T.D. All authors have approved the final version of the manuscript.

## Declaration of interests

The authors declare no competing interests.
